# Unveiling the Mystery of Peripartum Cardiomyopathy: A Traditional Review

**DOI:** 10.7759/cureus.10790

**Published:** 2020-10-04

**Authors:** Goodness C Chinweuba, Ian H Rutkofsky

**Affiliations:** 1 Obstetrics and Gynecology, Internal Medicine, California Institute of Behavioral Neurosciences & Psychology, Fairfield, USA; 2 Psychiatry, California Institute of Behavioral Neurosciences & Psychology, Fairfield, USA

**Keywords:** peripartum, cardiomyopathy, heart failure, pregnancy, outcomes of peripartum cardiomyopathy, diagnosis of peripartum cardiomyopathy, dyspnea, peripartum cardiomyopathy, dilated cardiomyopathy

## Abstract

Peripartum cardiomyopathy (PPCM) can be classified as a variant of dilated cardiomyopathy identified usually within the first five months of delivery or during the last month of pregnancy. This condition presents as systolic heart failure. PPCM affects thousands of women in the United States each year. Even though it was first noticed in the 1800s, its etiology remains unknown. This study aims to review the pathophysiology and management of PPCM and explore the possible outcomes of peripartum cardiomyopathy. PPCM can lead to maternal death if diagnosis or treatment is delayed. Diagnosing PPCM has been challenging because it can be misdiagnosed or perceived as a sign of pregnancy since most of the symptoms of PPCM strongly match those within the typical pregnancy continuum and postpartum cycle. Patients' implications are fatal and carry a high mortality rate when PPCM is misdiagnosed or treatment is delayed. To accurately identify PPCM, using echocardiography, the left ventricular end-diastolic size and the ejection fraction should be measured to determine the severity of PPCM. Managing peripartum cardiomyopathy involves using traditional treatments for heart failure. Therapeutic recommendations are made depending on the patient's status (pregnancy, breastfeeding, postpartum) while considering the drug-safety profiles before administration. Some other treatments have also been used to control PPCM depending on how severe it has become; for example, antiarrhythmics have been used to treat cardiac arrhythmias when they ensue. In extreme cases, mechanical assistance and transplantation could be required. Based on the proposed pathophysiology involving the 16kDA anti-angiogenic sub-fragment, bromocriptine may be used even though it still needs more assessment due to limited evidence.

Using PubMed as a major search resource, a thorough analysis of publications was carried out after incorporating this review's inclusion and exclusion criteria. A total of 455,141 publications were found using keywords and keyword combinations. With a careful selection of articles, 31 publications provided relevant information on our primary endpoint. All articles in this examination were chosen without limitation to the type of study, including clinical trials, review articles, meta-analyses, and so on. Our review suggests that, based on factors such as early detection and management, disease severity, ethnicity, and quality of patient care, patients with PPCM presented different outcomes and prognosis. However, despite PPCM's high mortality rate and its risk of recurrence, most patients tend to recover within six months of disease onset.

## Introduction and background

The incidence of peripartum cardiomyopathy (PPCM) varies geographically. In the United States, it can be estimated as one in 4000 live births affecting women each year while in African countries like Nigeria, there is a higher incidence, estimated as one in 100 live births per year [1]. The incidence of PPCM may be explained by older maternal age, an increased rate of fertility-assisted treatments, and a higher occurrence of maternal hypertensive disorders [2]. Peripartum cardiomyopathy, which is also called postpartum cardiomyopathy, is a rare form of heart disease that normally presents in the last month of pregnancy or the first five months postpartum in the absence of any other identifiable heart disease. To fully define PPCM, we should at least identify one of the following factors using echocardiography: left ventricular end-diastolic area size greater than 2.7cm/m^2^ of the body surface, ejection fraction less than 45%, or a reduced fractional shortening of less than 30% [3].

Peripartum cardiomyopathy is heart disease with multiple manifestations and unexplored pathogenesis; however, it shares the most similarities with dilated cardiomyopathy, which presents as a systolic dysfunction due to ventricular dilation. While it is believed that both heart diseases share similar pathological paths like impaired cardiac microvasculature and high oxidative stress, these deleterious effects in postpartum cardiomyopathy usually reverse within one year after delivery as compared to dilated cardiomyopathy [4].

The outcomes of PPCM has been proved to be strongly associated with ethnicity and race. Women from Africa have presented with more severe systolic dysfunctions and lesser cardiac ejection fraction. This scrutiny is believed to have caused less recovery as compared to non-African ethnicities [5]. The feared complication in one-third of patients with non-ischemic cardiomyopathies, such as peripartum cardiomyopathy, is sudden cardiac death due to ventricular tachyarrhythmia. Nonetheless, studies have shown that the earlier the ejection fraction of the left ventricle reduces, the higher the risk of ventricular arrhythmia [6-7].

In this evaluation, we aim to explore the possible outcomes and management of peripartum cardiomyopathy. We will also summarize the epidemiology and pathophysiology of PPCM.

## Review

Methods 

This review study was conducted using preferred reporting items for the literature review. All studies included in the review were selected without the restriction of study type, including investigation of clinical trials, systematic reviews, and meta-analyses. Studies were selected based on gender (female only) and clinical preference. There was no geographical restriction in this search. The search was performed with articles related to postpartum or peripartum cardiomyopathy, heart failure, and pregnancy. This search yielded many articles like case reports, meta-analyses, editorials, and review articles.

The inclusion criteria included (1) English language publications, (2) studies published within the last 10 years, and (3) studies strictly related to PPCM, pregnancy, and heart failure.

The exclusion criteria included (1) non-English language publications and (2) articles published more than 10 years ago.

Using parallel strategies to identify original research and review of articles from PubMed, data were collected using keywords. Table 1 shows the keywords and combinations of keywords used for the literature search.

**Table 1:** Keywords and combinations of keywords used for the literature search

Keywords/combinations of keywords	Database	Number of studies
Pregnancy	Pubmed	278451
Heart failure	Pubmed	120626
Cardiomyopathy	Pubmed	51,381
Peripartum	Pubmed	3,359
Peripartum cardiomyopathy	Pubmed	805
Diagnosis of postpartum cardiomyopathy	Pubmed	263
Outcomes of peripartum cardiomyopathy	Pubmed	256

After applying the inclusion and exclusion criteria using the keywords as a searching tool, 31 publications from PubMed were used for this review article.

Discussion

Epidemiology of Disease

Peripartum cardiomyopathy occurrence in the United States varies from one in 1000 to 4000 live births [8]. The occurrence spectrum is likely to represent the diverse population dynamics, description, and misrepresentation arising from lack of understanding or misdiagnosis [8]. A new nationwide inpatient report review of 64-million medical discharge reports from 1000 hospitals in 47 states shows 34219 cases of PPCM, with an occurrence of one in 968 births. Greater than half of the cases of PPCM arose in the southern United States, perhaps representing ethnic disparities. For example, the occurrence increased from 8.5 to 11.8 for 10000 live births from 2004 to 2011 and even from 2.3 to 4.5 per 10000 live births from a previous analysis from 1990 to 2002 [9]. The rising number may be due to factors like increased understanding and detection, increased maternal age, shifting demographics, and a high number of twin gestations. PPCM resulted in multiple maternal death during pregnancy as well as postpartum. PPCM was the leading factor (23%) in a demographic study of maternal cardiovascular deaths in California between 2002 and 2006 [10].

Pathophysiology of Peripartum Cardiomyopathy

Hemodynamic improvements in maternal circulation also begin in the first trimester, with a significant decrease in systemic vascular resistance and a subsequent rise in cardiac output [11]. Renin-angiotensin - aldosterone stimulation retains blood pressure and helps conserve salt and water while maternal systemic and renal arterial dilation maintains cardiovascular output [12]. Around the same period, vascular (predominantly arterial) stimulation is also powered by pregnancy hormones such as estrogen, progesterone, and relaxin. Heart remodeling contributes to a significant increase in left ventricular mass while angiogenesis increases [13-14]. During labor and delivery, cardiac output increases due to increased heart rate and preload caused by uterine contractions, elevated circulating catecholamines, and the auto-transfusion of 300-500 mL of blood from the fetus to the maternal bloodstream, which is consistent with labor during birth [11,13].

The etiology of PPCM is still a pending puzzle yet to be accurately solved. A hybrid 'two-hit' paradigm involving systemic angiogenic dysfunction and patients' vulnerability (predisposition) is thought to be critical in PPCM pathophysiology. Potential factors contributing to PPCM involve genetic predisposition, low levels of selenium, viral infections, stress-activated cytokines, inflammation, autoimmune reaction, inflammatory response to hemodynamic changes, oxidation, as well as the effects exerted by anti-angiogenic factors [15-16]. In particular, 16‐kDa, a smaller anti-angiogenic sub-fragment, induced by the oxidative stress of prolactin hormone, may trigger PPCM by causing endothelial injury [17-21]. MicroRNAs appear to be a study focus for heart failure; De Rosa et al. assessed plasma microRNAs in aortic and coronary venous sinuses in heart failure patients and control subjects [22]. A significant transcoronary gradient consistent with a significant cardiac release was observed in ischemic and non-ischemic heart failure patients with different microRNAs [22-23].

Genetics may also play a role in PPCM prevalence; genetically mediated dilated cardiomyopathy (DCM) may evolve during early adulthood and is often hard to distinguish from PPCM. Recent findings also support the notion that about 15%-20% of peripartum heart failure patients have cardiomyopathy-induced mutations, i.e., in genes such as titin, beta-myosin, myosin-binding protein C, lamin A/C, or sodium voltage-gated channel alpha subunit 5 (SCN5A). One hypothesis is that in gene-positive, phenotype-negative female patients with no clinical symptoms before pregnancy, the physiological stress encountered during pregnancy and delivery can uncover concealed DCM [24].

The incidence of mutations in patients with peripartum cardiomyopathy correlated with familial dilated cardiomyopathy genes indicates that these two diseases' clinical range may overlap. Van Spaendonck-Zwarts and colleagues reported five peripartum cardiomyopathy cases within 90 familial dilated cardiomyopathy cases. They also reported three peripartum cardiomyopathy patients with partial rehabilitation and first-degree dilated cardiomyopathy in their families, including one patient with c.149A > G, p. Gln50Arg.e. Other researchers reported familial mutations in the MYH7, SCN5A, and PSEN2 genes and spontaneous mutations in peripartum cardiomyopathy patients with the MYH6 and TNNT2 DCM genes. If indeed, these findings are accurate, the above results reflect an actual correlation between cardiac gene mutations and peripartum cardiomyopathy [19,25-26].

No particular potential causes have yet been established for peripartum cardiomyopathy. Nevertheless, black race, multiparity, maternal age > 30 years, twin gestations, a history of hypertension, preeclampsia, and eclampsia have also correlated with a more significant occurrence of peripartum cardiomyopathy, although no causal relationship has been established [26]. However, newer research has shown that the above findings may be genuinely related to peripartum cardiomyopathy.

Symptoms of Peripartum Cardiomyopathy

Shortly after delivery or when PPCM ensues, most likely in the first week post-delivery, common signs like orthopnea and paroxysmal nocturnal dyspnea are suggestive of heart failure. Usually, these symptoms are attributed to normal pregnancy, and this explains why the diagnosis of PPCM is usually missed in the early stages of the disease. The physical examination results include tachycardia, elevated jugular venous pressure, bilateral pulmonary crackles caused by pulmonary edema, a third heart sound (S3), and a displaced apical pulse. Severe cases may occur, which may warrant admission into the intensive care unit, presenting as acute respiratory failure or cardiogenic shock needing close monitoring [27].

Table 2 illustrates the difference as well as the similarities between typical signs of pregnancy and PPCM.

**Table 2:** Similarities and differences between the signs of pregnancy and PPCM * present, PND - paroxysmal nocturnal dyspnea, PPCM - peripartum cardiomyopathy

Signs	Pregnancy	PPCM
Dyspnea	*	*
Fatigue	*	*
Tachycardia/palpitations	*	*
Edema	*	*
Chest pain	*	*
Rales	*	*
S3 heart sounds	*	*
Hepatosplenomegaly	*	
Increased jugular venous pressure		*
Hemoptysis		*
PND/orthopnea	*	*

Evaluation of Peripartum Cardiomyopathy

The first steps in evaluating PPCM include general blood workup to evaluate signs such as anemia, electrolyte irregularities, endocrine disorders such as thyroid diseases, and renal or liver failure. Even though there are no specific diagnostic tests for PPCM, it remains a diagnosis of exclusion, which requires detailed investigation. Table 3 below illustrates the common diagnostic tests used in evaluating PPCM and the findings that can help rule in the condition.

**Table 3:** Common diagnostic tests used in evaluating PPCM and possible findings

Diagnostic test	Findings
Echocardiogram	Left ventricular ejection fraction less than 45%
Echocardiography	The most common sign is left ventricular hypertrophy with ST-T wave abnormalities
Cardiac biomakers	Elevation of brain natriuretic peptide
Radiography	Cardiomegaly

Treatment and Management of Peripartum Cardiomyopathy

PPCM is currently being treated in order to comply with the guidelines for heart failure in pregnancy. In the later phases of pregnancy, treatment modalities need to consider the patient's health and that of her fetus. Beta-blockers, thiazide diuretics, or loop diuretics can be initiated before delivery in certain PPCM patients, but this is done carefully and caution should be taken, as diuretic therapy can impair the blood supply to the placenta with possible fetal harm. Therefore, to prevent such cases, the lowest possible doses of diuretic therapy should be used. During the postpartum period, the standard heart failure therapy in PPCM, including beta-blockers, angiotensin-converting enzyme (ACE) inhibitors, angiotensin II receptor blockers (ARBS), mineralocorticoid receptor antagonists, and diuretics are recommended following delivery. Since there are no data on the use of inotropes like dobutamine in PPCM, knowing that catecholamines in such patients can provoke myocardial damage, intravenous infusion should be initiated in cases of profound hypotension in patients with PPCM. When the blood pressure and volume return to normal, inotropes can be slowly tapered and the standard guidelines used for patients with heart failure in pregnancy should be followed afterward. Even in patients with a severely depressed ejection fraction, early beta-blocker therapy has shown to be protective at very low doses [11].

Ivabradine, a selective If (funny channel) channel blocker, slows the heart rate by decreasing the sinus node's automaticity. It has been suggested that ivabradine decreases heart rate without further increasing the deleterious effects of congestive failure (CHF) in patients who already have CHF and an altered left ventricular function or have contraindications to beta-blockers. Ivabradine is beneficial in treating patients with left ventricular dysfunction and decompensated heart failure when comparing it to beta-blockers [28].

Atrial fibrillation, so far, has been the most common arrhythmia in peripartum cardiomyopathy patients. Some antiarrhythmic therapies have been used to salvage arrhythmias in PPCM patients, and amongst this list, quinidine and procainamide are reasonably safe to use in the puerperium, and for this reason, they have been used as the first-line medication for PPCM patients. Digoxin, a cardiac glycoside, can also be used as first-line therapy, as it has a positive inotropic effect and a negative chronotropic effect. In some refractory cases of atrial fibrillation, a permanent pacemaker and an implantable cardioverter-defibrillator will be required. As we discuss possible treatments, we should also remember that heart failure and PPCM predispose patients to thromboembolism. Anticoagulants like low molecular weight heparin should be added during pregnancy, and when the left ventricular ejection fraction is less than 0.30 in the postpartum period, warfarin can be initiated [27].

Bromocriptine, an agonist of the dopamine receptor with prolactin-blocking properties, was also found to be effective in PPCM patients. According to a study by Hakata et al., bromocriptine was reportedly useful in treating cardiomyopathy. It was speculated that prolactin over-secretion increased the amount of a 16-kDa antiangiogenic prolactin fragment, which, as mentioned above, has played a vital role as a pathological mediator of PPCM by adversely affecting myocardial micro-vascularization [8,27]. As a result, initiating bromocriptine therapy, an anti-prolactin, may decrease the level of the 16-kDa fragment and help improve the general cardiac function [27,29].

Outcomes of the Disease

Patients with peripartum cardiomyopathy have a strong tendency to recover. At the time of diagnosis, the left ventricular ejection (LVEF) fraction correlates with improved survival rates and higher rates of complete recovery [30]. Deciding on when to discontinue treatment plays a crucial role in a PPCM patient's general outcome because, most times, it is not so clear if and when a patient has recovered completely. Nonetheless, it is best to wait for the normalization of left ventricular (LV) function before stopping medications, and women should also be informed that they are still at a high risk of recurrence even if their LV function has returned to the normal state. However, PPCM could still lead to a severe and sometimes persistent decrease in LV function or even life-threatening complications, as the long-term outcomes have not yet been established with clarity [29,31].

Long-term results vary amongst patients. With routine medical care, up to 50% of patients with PPCM show improvements; however, 25% of patients end up developing chronic heart failure while the remaining dies during the disease course. If the ejection fraction remains low, pregnancy should be avoided because survival rates appear to be low in such circumstances. Patients who desire to get pregnant in the future should wait for at least five years after the initial ejection fraction has normalized [27].

Limitations 

While we were compiling pieces of information for this review, some limitations were encountered. PPCM is a rare condition and we were unable to find literature that explained its etiology accurately. A way to reduce this limitation would be the establishment of further research studies to identify the cause(s) of PPCM. Another limitation encountered was that of finding literature that had specific diagnostic tests for PPCM. PPCM is ideally a diagnosis of exclusion and the subsequent limitation can be reduced by further researching specific diagnostic parameters. PPCM is an important condition that affects both mother and child and investigating these limitations will lead to improved fetal and maternal outcomes.

## Conclusions

Peripartum cardiomyopathy is an uncommon yet highly fatal condition with unexplained etiology. The diagnosis of peripartum cardiomyopathy requires early recognition of signs and symptoms, including the early initiation of medical therapy to prevent further complications and preserve life. Peripartum cardiomyopathy treatment can mainly strive to manage heart failure signs by therapeutic approaches used in treating heart failure. In some cases, specific targeted therapies like bromocriptine can be considered; however, further clinical evaluation is needed before they can be implemented fully. The outcome and prognosis are best if PPCM is diagnosed and managed early. It is also crucial that clinicians are adequately acquainted with PPCM, and in doing so, they should always have it in mind as a potential diagnosis when treating dyspneic patients within the PPCM timeframe, as this can be a lethal condition. Further research is required to understand the complexity of PPCM in the near future.
